# Emission from the working and counter electrodes under co-reactant electrochemiluminescence conditions†

**DOI:** 10.1039/d1sc01236c

**Published:** 2021-06-25

**Authors:** Natasha S. Adamson, Ashton G. Theakstone, Lachlan C. Soulsby, Egan H. Doeven, Emily Kerr, Conor F. Hogan, Paul S. Francis, Lynn Dennany

**Affiliations:** School of Life and Environmental Sciences, Faculty of Science, Engineering and Built Environment, Deakin University Waurn Ponds 3216 Australia paul.francis@deakin.edu.au; Institute for Frontier Materials, Deakin University Waurn Ponds 3216 Australia emily.kerr@deakin.edu.au; Centre for Regional and Rural Futures, Faculty of Science, Engineering and Built Environment, Deakin University Waurn Ponds 3216 Australia; Department of Chemistry and Physics, La Trobe Institute for Molecular Science, La Trobe University Melbourne VIC 3086 Australia; WestCHEM, Department of Pure and Applied Chemistry, University of Strathclyde, Technology and Innovation Centre 99 George Street Glasgow G1 1RD UK lynn.dennany@strath.ac.uk

## Abstract

We present a new approach to explore the potential-dependent multi-colour co-reactant electrochemiluminescence (ECL) from multiple luminophores. The potentials at both the working and counter electrodes, the current between these electrodes, and the emission over cyclic voltammetric scans were simultaneously measured for the ECL reaction of Ir(ppy)_3_ and either [Ru(bpy)_3_]^2+^ or [Ir(df-ppy)_2_(ptb)]^+^, with tri-*n*-propylamine as the co-reactant. The counter electrode potential was monitored by adding a differential electrometer module to the potentiostat. Plotting the data against the applied working electrode potential and against time provided complementary depictions of their relationships. Photographs of the ECL at the surface of the two electrodes were taken to confirm the source of the emissions. This provided a new understanding of these multifaceted ECL systems, including the nature of the counter electrode potential and the possibility of eliciting ECL at this electrode, a mechanism-based rationalisation of the interactions of different metal-complex luminophores, and a previously unknown ECL pathway for the Ir(ppy)_3_ complex at negative potentials that was observed even in the absence of the co-reactant.

## Abstract 2

Exploration of potential-dependent, multi-colour co-reactant electrochemiluminescence from multiple luminophores at the working and counter electrodes reveals new pathways to emission.

## Graphical abstract

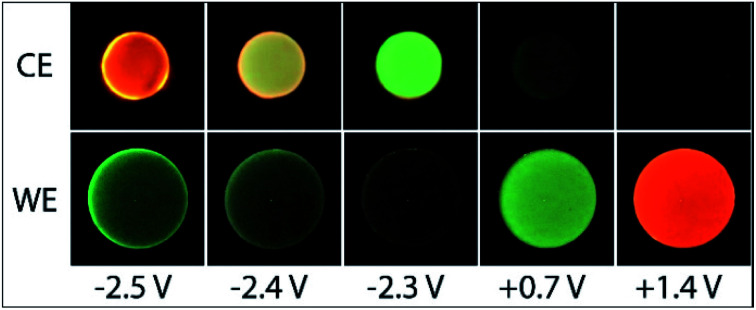

## Introduction

Unlike spectroscopic modes of detection that require an excitation light source, chemiluminescence is elicited by chemical reactions and thus can be measured against a dark background.^1,2^ Electrochemical initiation of these light producing reactions (*i.e.* electrochemiluminescence or electrogenerated chemiluminescence; ECL) provides not only greater spatial and temporal control of the emission but also the ability to manipulate the competing excitation pathways, by controlling the timespan and magnitude of the applied potentials.^3–5^ This has been increasingly exploited to simultaneously or selectively excite multiple luminophores in multi-colour and/or potential-resolved ECL systems.^6–12^

In traditional ECL experiments, the emission is generally assumed to emanate near the surface of the working electrode, with occasional observations of ECL at the counter electrode dismissed as an unwanted interference.^5,13^ Moreover, conventional ECL detectors such as a photomultiplier tube (PMT) or charge coupled device (CCD) spectrometer measure the cumulative ECL from the electrochemical cell.^14^ Such detectors do not provide spatial resolution and, therefore, the source of the emission (*i.e.* if ECL is emanating from the working or counter electrode) is not evident. Contributions from reactions at the counter electrode can therefore be misinterpreted, which may be more common than previously realised.^15,16^ Recent advances in spatially resolved ECL sensing strategies employing digital cameras have allowed for the simultaneous detection of ECL at both the working and counter electrodes.^17,18^

Initiation of ECL from multiple luminophores often necessitates application of a wider range of potentials at the working electrode.^6^ This in turn places greater electrochemical demands at the counter electrode, which, in conjunction with the presence of more diverse electroactive species, increases the likelihood of ECL at this electrode. Although this increases the possibility of interferences, it also provides opportunities to design simultaneous spatially-resolved ECL systems, where ECL emanates from both the working and counter electrodes.^17^

The most commonly used multi-colour co-reactant ECL system comprises tris(2,2′-bipyridine)ruthenium(ii) ([Ru(bpy)_3_]^2+^) (or a dicarboxamide or dicarboxylate derivative) and tris(2-phenylpyridinato)iridium(iii) (Ir(ppy)_3_), with tri-*n*-propylamine (TPrA) as the co-reactant, in acetonitrile.^6,19–25^ This combination of ECL luminophores was initially examined using conventional three-electrode electrochemical cells for the development of multiplexed ECL labelling strategies, where multiple analytes are detected using luminophores that emit different colours and/or are excited at different applied potentials.^19,20^ Recently, the potential-dependent emission of different luminophores has also been exploited for multi-colour imaging or reporting of electrode potentials in bipolar ECL systems.^22,23,25^

Herein we characterise the potential-dependent multi-colour co-reactant ECL from the [Ru(bpy)_3_]^2+^, Ir(ppy)_3_ and TPrA system at both the working and counter electrode in a three-electrode cell. To understand the ECL processes, we simultaneously measured the potentials at the working and counter electrodes, the current between these electrodes, and the ECL emission over cyclic voltammetric scans.

## Results and discussion

### Co-reactant ECL with a single luminophore

The electrochemistry and spectroscopy of [Ru(bpy)_3_]^2+^ and Ir(ppy)_3_ have been well characterised (Table 1).^13,26–30^ To ensure reproducible visualisation of the ECL emission, we used disk electrodes for both the working (glassy carbon) and counter (platinum). The magnitude of the potential at the counter electrode was augmented by its size (2 mm i.d.) relative to the working electrode (3 mm i.d.). A counter electrode with relatively large surface area, such as a metal wire, basket, flag or mesh, is often used in ECL experiments to minimise the effects of interfering reactions at that electrode by decreasing the current density.^31,32^ Previous imaging of the source of emission in ECL cells, however, has suggested that the ECL active area of the electrode may be localised on a portion of the metal wire surface, probably due to geometric factors.^18^

**Table 1:** Electrochemical and spectroscopic data

	*E* ^0^′(ox)^a^/V	*E* ^0^′(red)^a^/V	*λ*_max_ b/nm	*E*_00_ c/eV
[Ru(bpy)_3_]^2+^	1.25	−1.37, −1.56, −1.82, −2.45^d^	620	2.14
Ir(ppy)_3_	0.68	−2.32, −2.57,^e^ −2.81^e^	520	2.51
[Ir(df-ppy)_2_(ptb)]^+^	1.56	−1.80, −2.23, −2.51^d^	454, 482	2.77
TPrA	0.89^d^			
TPrA˙	−1.7^f^			

a Potential (V) *vs.* Ag/AgCl.

b From spectra corrected for the change in instrument sensitivity across the examined wavelength range.^26,27,33^

c Excited state energies approximated from emission spectra at 77 K or 85 K.^26,28,33,34^

d Irreversible (*E*_p_).

e Estimated from difference in potentials of the 1st, 2nd and 3rd reductions measured in other solvents.^35^

f Estimated by Lai and Bard.^36^

**Fig. 1:**
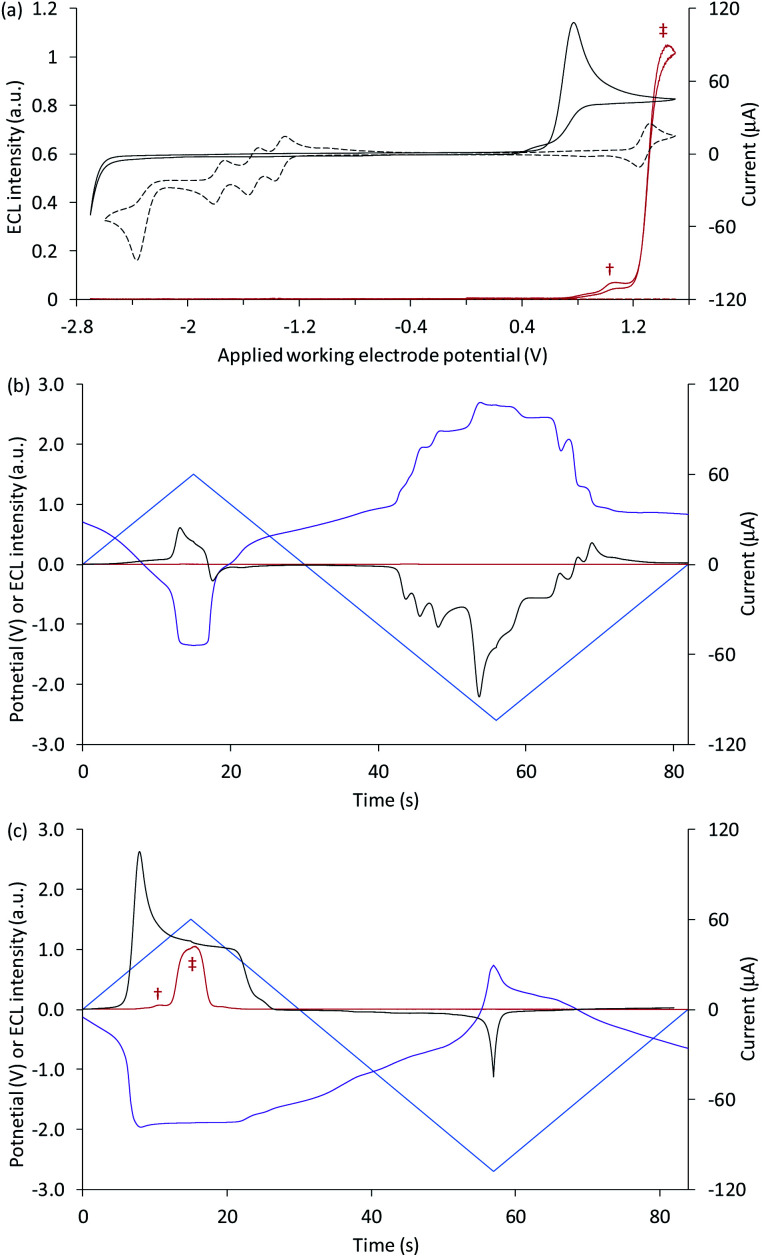
(a) Cyclic voltammograms (black plots) and corresponding ECL intensities (red plots) for 5 µM [Ru(bpy)_3_]^2+^ with 10 mM TPrA (solid plots) and 1 mM [Ru(bpy)_3_]^2+^ (dashed plots). (b and c) The potential applied at the working electrode (blue plots), potential measured at the counter electrode (purple plots), ECL intensity (red plots), and current (black plots), *vs.* time, during one cycle of a CV experiment, for (b) 1 mM [Ru(bpy)_3_]^2+^, and (c) 5 µM [Ru(bpy)_3_]^2+^ with 10 mM TPrA. All solutions contain 0.1 M TBAPF_6_ electrolyte in ACN and were degassed for 10 min prior to analysis (scan rate 0.1 V s^−1^). † ‘First-wave’ co-reactant ECL (reactions (1)–(5)). ‡ ‘Second-wave’ co-reactant ECL (predominantly reactions (1)–(3), and (5)–(8)). The ECL intensity from the cell was measured using a PMT. The location of the emission (*i.e.* at the working or counter electrode) at different applied potentials was verified by photography (for example, see Fig. 2a).

Fig. 1a shows a CV of 1 mM [Ru(bpy)_3_]^2+^ (dashed black plot), with peaks that can be assigned to the reversible oxidation of the metal centre, three reversible ligand reductions and a fourth, irreversible reduction (Table 1). The overlaid CV of 5 µM [Ru(bpy)_3_]^2+^ with 10 mM TPrA is dominated by the broad irreversible oxidation peak of the co-reactant at 0.7–0.8 V *vs.* Ag/AgCl (solid black plot). The standard potential for the TPrA˙^+^/TPrA couple (reaction (1)) has been estimated as 0.9 V *vs.* Ag/AgCl.¶¶Lai and Bard reported the standard potentials for the oxidation of TPrA and TPrA˙ as 0.9 V and −1.7 V *vs.* SCE, respectively.^36^ We have referenced the potentials to Ag/AgCl for consistency with our other values (0 V *vs.* Ag/AgCl (3.5 M KCl) = −0.039 V *vs.* SCE).^36^ The aminium radical cation TPrA˙^+^ rapidly deprotonates to form an α-aminoalkyl carbon-centred radical, denoted TPrA˙ (reaction (2)).^37,38^

1 TPrA − e^−^ → TPrA˙^+^

2 TPrA˙^+^ → TPrA˙ + H^+^

Under these experimental conditions, we observed weak ECL between 0.8 V and 1.2 V (*vs.* Ag/AgCl), and a more intense emission above 1.2 V *vs.* Ag/AgCl (red plot, Fig. 1a). These emissions can be confidently ascribed to the first- and second-wave oxidative-reduction co-reactant ECL mechanisms established for this system.^39^ Below the potential at which the ruthenium complex is oxidised, the light-producing pathway comprises reactions (1)–(5) (where M is [Ru(bpy)_3_]^2+^), but at potentials at which the metal complex is oxidised, reactions (6)–(8) (and to a lesser extent, reaction (9)) can also contribute.

3 M + TPrA˙ → M^−^ + other products

4 M^−^ + TPrA˙^+^ → M* + TPrA

5 M* → M + *hν*

6 M − e^−^ → M^+^

7 M^+^ + TPrA → M + TPrA˙^+^

8 M^+^ + TPrA˙ → M* + other products

9 M^+^ + M^−^ → M* + M

To explore the electrochemical processes underpinning the co-reactant ECL of [Ru(bpy)_3_]^2+^ and TPrA at both working and counter electrode, we monitored the potential at the counter electrode using a differential electrometer module added to the potentiostat. We previously used this instrumental configuration to measure the counter electrode potential upon application of chronoamperometric pulses at the working electrode.^17,18^ but here it is exploited for the first time to explore ECL initiated by cyclic voltammetry. This enabled the potential at the two electrodes, the current, and the ECL intensity to be plotted *versus* time, as shown in Fig. 1b for 1 mM [Ru(bpy)_3_]^2+^, and Fig. 1c for 5 µM [Ru(bpy)_3_]^2+^ with 10 mM TPrA. Extending these plots over successive CV scans (Fig. S1†) shows the reproducibility of the changes in the electrode potentials and current. Moreover, the relationship between the counter electrode potential and the applied working electrode potential is shown in Fig. S2.†

The potential at the counter electrode is governed by the product of the current and the so-called compensated resistance dropped across the cell.^40^ It is therefore dependent not only on the applied potential, but also the nature and concentration of the electroactive species at both electrodes. In Fig. 1b, when cathodic potentials are applied (when the blue plot is below zero), the counter electrode (purple plot) reaches potentials (up to 2.22 V *vs.* Ag/AgCl) far above those required to oxidise [Ru(bpy)_3_]^2+^. As expected, in the absence of co-reactant, no ECL was detected (red plot).||||We detected weak ECL after repeated CV cycles due to annihilation of electrochemically oxidised and reduced forms (reaction (9)).

**Fig. 2:**
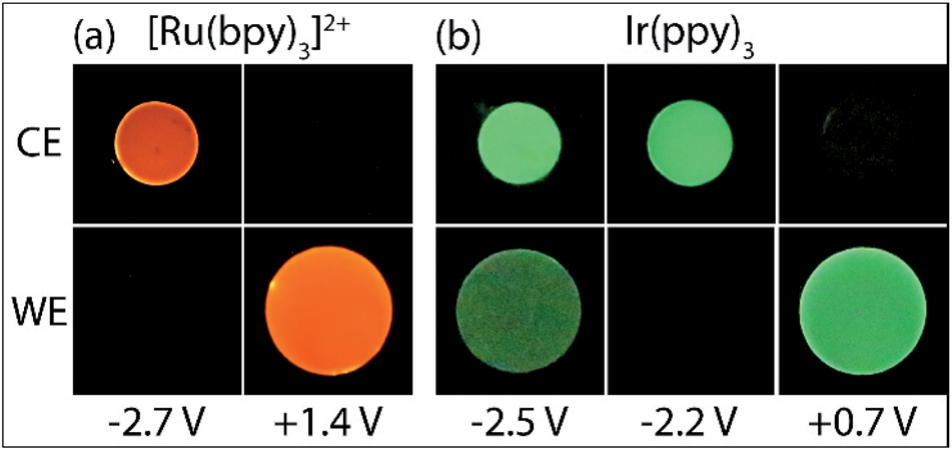
Photographs of the ECL of (a) 5 µM [Ru(bpy)_3_]^2+^ and 10 mM TPrA, or (b) 0.2 mM Ir(ppy)_3_ and 10 mM TPrA, with 0.1 M TBAPF_6_ in acetonitrile, at the working electrode (WE) and counter electrode (CE), upon application of different working electrode potentials.

As shown in Fig. 1c, with the addition of TPrA, the same cathodic scan range at the working electrode (blue plot) is maintained through comparatively modest counter electrode potentials of up to 0.73 V *vs.* Ag/AgCl, near the onset of the weak first-wave co-reactant ECL at the working electrode (Fig. 1a). The magnitude of anodic potentials at the counter electrode was raised to 0.79 V *vs.* Ag/AgCl by scanning cathodic potentials at the working electrode without prior scanning of anodic potentials, and a weak ECL emission was detected. Moreover, when applying a chronoamperometric pulse at −2.7 V *vs.* Ag/AgCl at the working electrode, the counter electrode potential extends beyond that required to oxidise the luminophore (Fig. S3a†), with an initial spike to 1.28 V *vs.* Ag/AgCl. As shown in Fig. 2a, under these conditions, we observe orange ECL from anodic reactions (oxidative-reduction co-reactant ECL) at the counter electrode.^15,18^

**Fig. 3:**
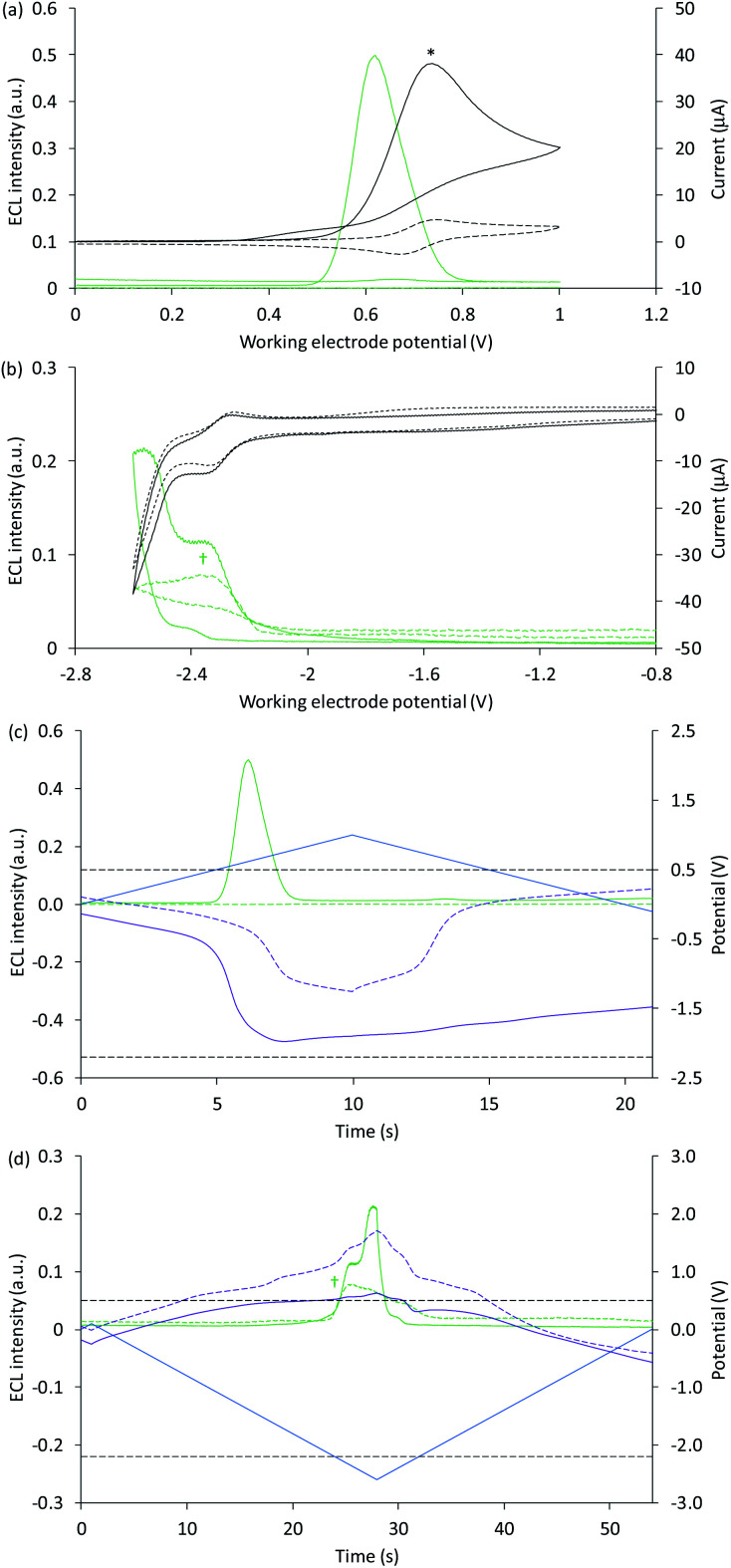
(a and b) Cyclic voltammograms (black plots) and corresponding ECL intensities (green plots) for 0.2 mM Ir(ppy)_3_ with (solid plots) and without (dashed plots) 10 mM TPrA, when applying (a) positive or (b) negative potentials at the working electrode. (c and d) Potential measured at the counter electrode (purple plot), and ECL intensity (green plot), over time, when scanning (c) positive or (d) negative potentials (blue plot), for 0.2 mM Ir(ppy)_3_ with (solid plots) and without (dashed plots) 10 mM TPrA. The dashed grey horizontal lines in Fig. 2c and d indicate the potentials at which the on-set of ECL was observed in Fig. 2a and b. All solutions contain 0.1 M TBAPF_6_ electrolyte in ACN and were degassed for 10 min prior to analysis (scan rate 0.1 V s^−1^). To show the plots on the same scales, the current for 0.2 mM Ir(ppy)_3_ with 10 mM TPrA at positive potentials (*) was divided by five, and the ECL intensity in the absence of TPrA at negative potentials (†) was multiplied by 10. See Fig. S4† for the change in current overlaid on (c) and (d). See Fig. S5† for the same plots extended over three CV scans. In an alternative representation, the change in counter electrode potential (and ECL intensity) against the applied working electrode potential is shown in Fig. S6.†

The Ir(ppy)_3_ complex also exhibits a reversible oxidation attributed to the metal centre, but at a considerably lower potential than that of [Ru(bpy)_3_]^2+^ (Table 1).^30^ ECL was predominantly observed between 0.5 V and 0.8 V *vs.* Ag/AgCl (Fig. 2b and 3a). As this complex is considerably more reductive than [Ru(bpy)_3_]^2+^, the light producing pathway for oxidative-reduction co-reactant ECL with TPrA is limited to reactions (1), (2), (6), (8), (5) (and the reverse of reaction (7)), where M is now Ir(ppy)_3_.^41^

The ‘switch-off’ of the green ECL at moderate to high positive applied potentials has been ascribed to oxidative quenching of [Ir(ppy)_3_]* by the co-reactant radical cation (reaction (10)).^42^

10 M* + TPrA˙^+^ → M^+^ + TPrA

A negative potential scan for Ir(ppy)_3_ shows a single, reversible ligand reduction (Fig. 3b), although second and third reduction steps have been observed in other solvents.^35^ The counter electrode attained anodic potentials sufficient for the oxidative-reduction co-reactant ECL of Ir(ppy)_3_ upon application of lower magnitude cathodic potentials at the working electrode (−2.2 V *vs.* Ag/AgCl; Fig. 2b and 3d), considerably lower than that required for the analogous ECL of [Ru(bpy)_3_]^2+^, as expected.

Surprisingly, when examining the images of the co-reactant ECL of Ir(ppy)_3_ with potentials applied as chronoamperometric pulses, we observed light at the working electrode under large negative potentials (Fig. 2b). Similarly, a faint emission emanated from the counter electrode upon application of positive potentials. The mechanism of the ECL under cathodic reaction conditions is currently unknown. It is observed at potentials beyond those required to reduce the luminophore (at the edge of the electrochemical window of the solvent) and is observed in the absence of TPrA (Fig. 3b and d, dashed green plot). We therefore tentatively postulate a pathway in which high energy species generated by reduction of the solvent or electrolyte^43–45^ serve as the co-reactant intermediates of a reductive-oxidation ECL pathway.**We repeated this experiment using DMF instead of ACN as the solvent and observed a less intense emission at the working electrode upon application of large negative potentials, in the absence of TPrA co-reactant, as shown in Fig. S12.†

### Co-reactant ECL with a multiple luminophores

With this instrumental approach, we examined the ECL of a mixture of Ir(ppy)_3_ and [Ru(bpy)_3_]^2+^ with TPrA co-reactant. As in most previous applications of this and closely related systems, a higher concentration of Ir(ppy)_3_ than [Ru(bpy)_3_]^2+^ was employed to compensate for the difference in their co-reactant ECL efficiencies.^20–22,25^

**Fig. 4:**
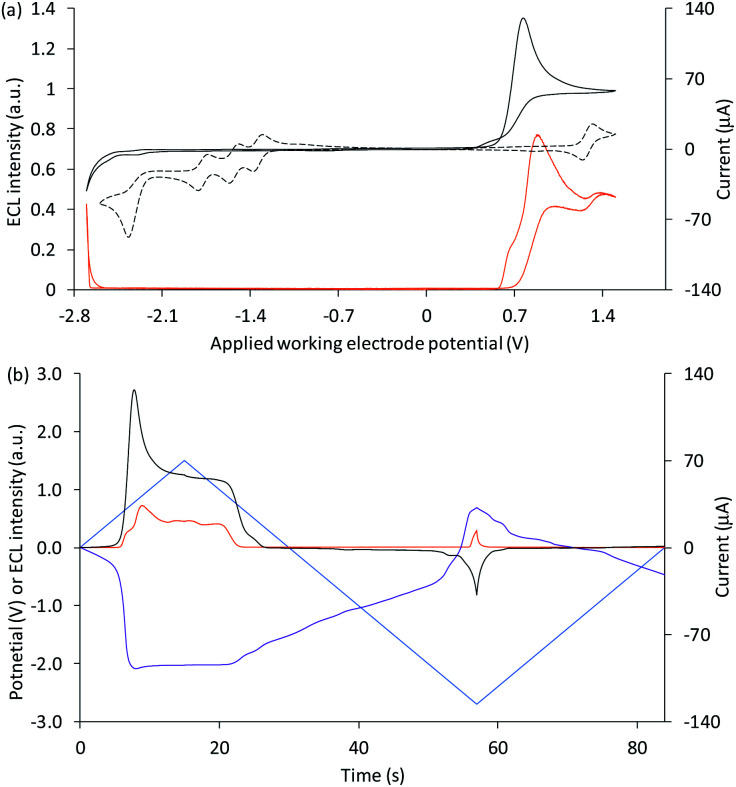
(a) Cyclic voltammogram (black plot) and corresponding ECL intensity (orange plot) for 0.1 mM Ir(ppy)_3_ and 5 µM [Ru(bpy)_3_]^2+^ with 10 mM TPrA, and the cyclic voltammogram (dashed black plot) for 1 mM [Ru(bpy)_3_]^2+^. (b) Potential measured at the counter electrode (purple plot), ECL intensity (orange plot) and current (black plot), over time, during one cycle of the cyclic voltammetry shown in Fig. 3a for 0.1 mM Ir(ppy)_3_ and 5 µM [Ru(bpy)_3_]^2+^ with 10 mM TPrA. All solutions contain 0.1 M TBAPF_6_ electrolyte in ACN and were degassed for 10 min prior to analysis (scan rate 0.1 V s^−1^). The change in counter electrode potential (and ECL intensity) against the applied working electrode potential is shown in Fig. S7.†

**Fig. 5:**
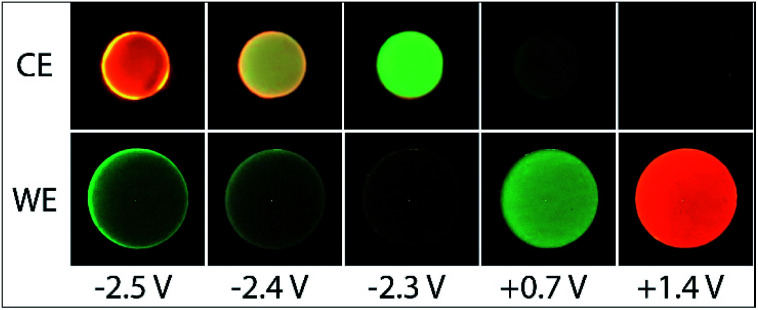
Photographs of the ECL of 5 µM [Ru(bpy)_3_]^2+^, 0.1 mM Ir(ppy)_3_ and 10 mM TPrA, with 0.1 M TBAPF_6_ in acetonitrile, at the working electrode (WE) and counter electrode (CE), upon application of different working electrode potentials.

The ECL profiles of this multi-luminophore system (Fig. 4 and 5) exhibited the combined characteristics of the two components with additional features arising from their interaction. In previous reports of the co-reactant ECL of Ir(ppy)_3_ and either a dicarboxamide^21,42^ or dicarboxylate^46^ derivative of [Ru(bpy)_3_]^2+^, scanning positive potentials elicited first the green and then the red emission of the respective luminophores, with a region of lower intensity in between (where the first-wave ECL of the Ru complex was observed).

Applications of the co-reactant ECL of Ir(ppy)_3_ and the parent [Ru(bpy)_3_]^2+^ complex,^22,23,25^ however, indicate much greater overlap of luminophore emissions. This can in part be attributed to the lower potential at which [Ru(bpy)_3_]^2+^ is oxidised compared to the two derivatives (Table S1†),^47^ which results in an earlier on-set of the intense second-wave ECL of the ruthenium complexes. However, as shown in Fig. 4, we observed the greatest ECL intensity from Ir(ppy)_3_ and [Ru(bpy)_3_]^2+^ (with TPrA co-reactant) at ∼0.9 V *vs.* Ag/AgCl (between the *E*^0^′(ox) of the luminophores; Table 1). At this potential, Ir(ppy)_3_ is oxidised, but the excited state [Ir(ppy)_3_]* subsequently generated upon reaction with TPrA˙ (reaction (8)) is quenched (reaction (10)), as seen in Fig. 3a. [Ru(bpy)_3_]^2+^ is not oxidised at 0.9 V *vs.* Ag/AgCl, but is reduced by TPrA˙ (reaction (3)) leading to the first-wave ECL (*via* reaction (4)). This emission is relatively weak under these conditions, as shown in Fig. 1a.

Considering the species available, the unexpectedly intense co-reactant ECL from [Ru(bpy)_3_]^2+^ at ∼0.9 V *vs.* Ag/AgCl in this system can be assigned to reaction (11). This reaction has previously been reported^48^ in the mixed annihilation ECL of Ir(ppy)_3_ and [Ru(bpy)_3_]^2+^ in acetonitrile (in the absence of any co-reactant), when applying alternating potentials sufficient to oxidise only Ir(ppy)_3_ and reduce only [Ru(bpy)_3_]^2+^.

11 [Ir(ppy)_3_]^+^ + [Ru(bpy)_3_]^+^ → Ir(ppy)_3_ + [Ru(bpy)_3_]^2+^*

Photographs of the co-reactant ECL from this multi-luminophore system upon application of 0.9 V *vs.* Ag/AgCl confirmed that the predominant source of emission under these conditions was [Ru(bpy)_3_]^2+^ (Fig. S8†).

In contrast, the dicarboxamide and dicarboxylate derivatives of [Ru(bpy)_3_]^2+^ are reduced at lower magnitude potentials (Table S1†) and despite the lower energies of their ^3^MLCT excited states, the analogous pathways to their emitting species *via* reaction with [Ir(ppy)_3_]^+^ (reaction (12)) are endergonic (Table S2†).

12 [Ir(ppy)_3_]^+^ + [Ru(bpy)_2_(L)]^+^ → Ir(ppy)_3_ + [Ru(bpy)_2_(L)]^2+^*

Application of negative electrochemical potentials to the solution of [Ru(bpy)_3_]^2+^, Ir(ppy)_3_ and TPrA elicited green emission at the working electrode (at potentials beyond −2.3 V *vs.* Ag/AgCl), which was detected from Ir(ppy)_3_ alone (Fig. 3b and d). This emission was not prominent on the scale used to quantify the ECL at 0.6–1.5 V *vs.* Ag/AgCl in Fig. 4, but can be clearly seen in the photographs (Fig. 5). The more intense ECL beyond −2.6 V *vs.* Ag/AgCl (Fig. 4a) arises from the oxidative-reduction co-reactant ECL of Ir(ppy)_3_ at the counter electrode, as its anodic potential exceeds that required for the on-set of this emission (Fig. 4b). As noted earlier, the chronoamperometric experiments used for the photographs result in greater magnitude potentials at the counter electrode and under these conditions, the oxidative-reduction co-reactant ECL of both Ir(ppy)_3_ and [Ru(bpy)_3_]^2+^ were observed (Fig. 5). In this region, the potential at the counter electrode changed rapidly with small changes to the applied potential at the working electrode (for example see Fig. S10†).

**Fig. 6:**
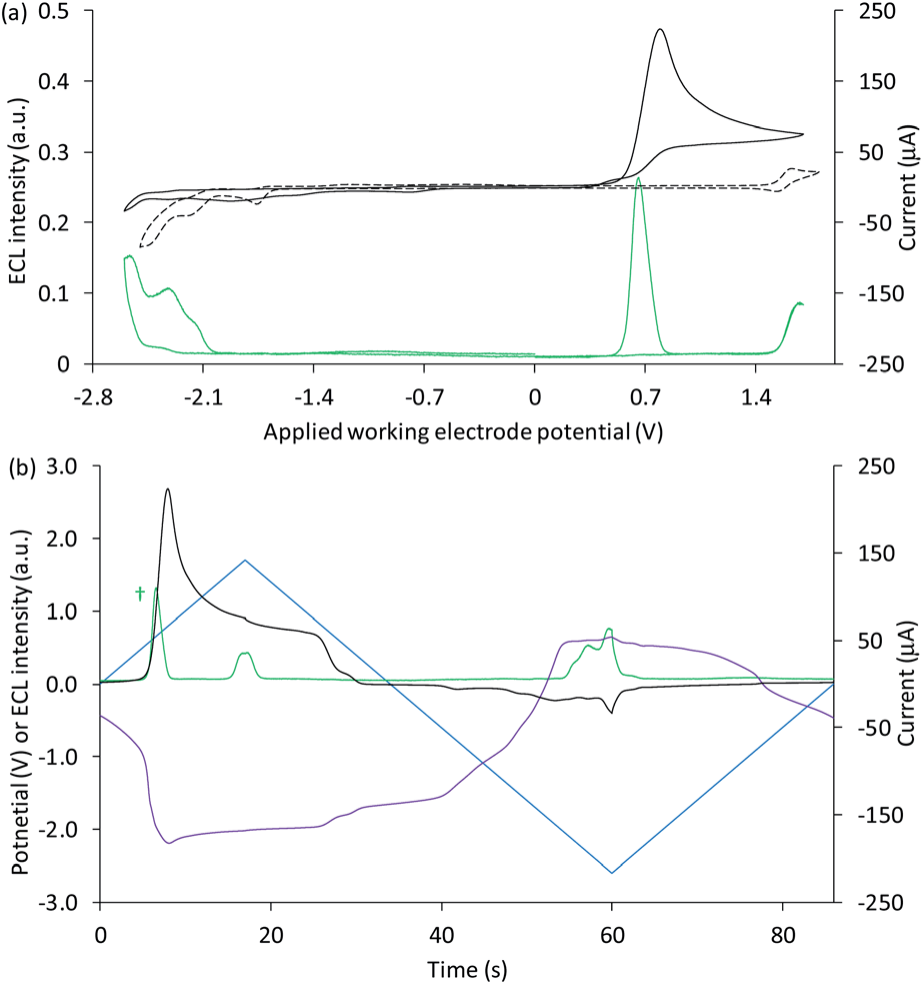
(a) Cyclic voltammogram (black plot) and corresponding ECL intensity (green plot) for 0.1 mM Ir(ppy)_3_ and 40 µM [Ir(df-ppy)_2_(ptb)]^+^ with 10 mM TPrA, and the cyclic voltammogram (dashed black plot) for 1 mM [Ir(df-ppy)_2_(ptb)]^+^. (b) Potential measured at the counter electrode (purple plot), ECL intensity (green plot) and current (black plot), over time, during one cycle of the cyclic voltammetry shown in (a) for 0.1 mM Ir(ppy)_3_ and 40 µM [Ir(df-ppy)_2_(ptb)]^+^ with 10 mM TPrA. All solutions contain 0.1 M TBAPF_6_ electrolyte in ACN and were degassed for 10 min prior to analysis (scan rate 0.1 V s^−1^). For clarity the ECL intensity in (b) (†) has been multiplied by 5. The change in counter electrode potential (and ECL intensity) against the applied working electrode potential is shown in Fig. S9.†

**Fig. 7:**
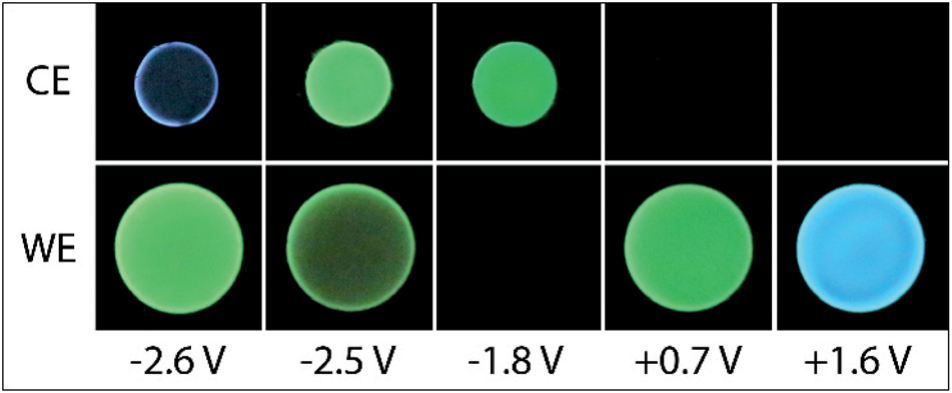
Photographs of the ECL of 40 µM [Ir(df-ppy)_2_(ptb)]^+^, 0.1 mM Ir(ppy)_3_ and 10 mM TPrA, with 0.1 M TBAPF_6_ in acetonitrile, at the working electrode (WE) and counter electrode (CE), upon application of different working electrode potentials.

We next examined the co-reactant ECL of Ir(ppy)_3_ combined with a higher energy (blue light) emitter, [Ir(df-ppy)_2_(ptb)]^+^. This complex has been identified as a promising blue electrochemiluminophore^33,49^ and utilised in multi-luminophore annihilation ECL systems.^48,50,51^ Various closely related high-energy luminophores (*e.g.*, Ir(df-ppy)_3_,^42,46^ Ir(df-ppy)_2_(pic),^8,52^ [Ir(df-ppy)_2_(ptp)]^+^,^46^ [Ir(df-ppy-CF_3_)_2_(ptb)]^+^,^9^ and [Ir(df-ppy-CF_3_)_2_(dtb-bpy)]^+^)^24^ have also been used in multi-luminophore annihilation and co-reactant ECL systems. As shown in Fig. 6, when increasingly positive potentials are applied to working electrode in a solution of Ir(ppy)_3_, [Ir(df-ppy)_2_(ptb)]^+^ and TPrA, two distinct emission bands were observed, corresponding to the oxidation potentials of the two luminophores (Table 1). The characteristic green and blue emissions at these potentials are shown in Fig. 7.

Unlike the system containing [Ru(bpy)_3_]^2+^, no significant emission was observed between these two bands, due to the efficient quenching of Ir(ppy)_3_ at high overpotentials (reaction (10)),^42^ and the absence of the first-wave co-reactant ECL pathway for [Ir(df-ppy)_2_(ptb)]^+^.^33^ Considering the degree of error in estimating the reduction potential of TPrA˙,^36^ we cannot rule out its reduction of [Ir(df-ppy)_2_(ptb)]^+^ (reaction (3)). Nevertheless, subsequent reaction of [Ir(df-ppy)_2_(ptb)]^0^ with either TPrA˙^+^ (reaction (4)) or [Ir(ppy)_3_]^+^ (analogous to reaction (11)) is not sufficiently energetic to attain the electronically excited [Ir(df-ppy)_2_(ptb)]^+^*. The reaction with [Ir(ppy)_3_]^+^, however, can generate [Ir(ppy)_3_]* (reaction (13)),^50^ but in this region it is efficiently quenched.

13 [Ir(ppy)_3_]^+^ + [Ir(df-ppy)_2_(ptb)]^0^ → [Ir(ppy)_3_]* + [Ir(df-ppy)_2_(ptb)]^+^

Applying negative potentials elicits ECL peaks at −2.3 V and −2.5 V *vs.* Ag/AgCl (Fig. 6), which can be assigned to the postulated reductive-oxidation pathway of Ir(ppy)_3_ at the working electrode, and the oxidative-reduction co-reactant ECL of Ir(ppy)_3_ and TPrA at the counter electrode. As previously noted, the chronoamperometry experiments used to obtain the photographs (Fig. 7) increase the magnitude of the initial peak in the corresponding counter electrode potentials and the oxidative-reduction co-reactant ECL of Ir(ppy)_3_ and then [Ir(df-ppy)_2_(ptb)]^+^ with TPrA are observed. The large gap between luminophore emissions of this system at the working electrode did not occur at the counter electrode (Fig. 7). Similarly, when large negative potentials were applied to the system containing [Ru(bpy)_3_]^2+^, the green emission of Ir(ppy)_3_ persisted further into the region in which the orange emission became intense (Fig. 5). These differences can be rationalised by the large changes in counter electrode potential relative to the applied working electrode potential towards the edge of the electrochemical window of the solvent (for example see Fig. S3 and S10†), and the efficiency of TPrA oxidation of the different electrode materials,^53–55^ which determine the rates of formation and quenching of [Ir(ppy)_3_]*.

## Experimental

### Materials and methods

Tris(2,2′-bipyridine)ruthenium(ii) hexafluorophosphate ([Ru(bpy)_3_](PF_6_)_2_), tris(2-phenylpyridinato)iridium(iii) (Ir(ppy)_3_), tri-*n*-propylamine (TPrA), *N*,*N*-dimethylformamide (DMF, Biotech grade, less than 8 ppm free amines as dimethylamine), and tetrabutylammonium hexafluorophosphate (TBAPF_6_) were purchased from Merck (NSW, Australia). Acetonitrile (ACN) was purchased from ChemSupply Australia. Bis[3,5-difluoro-2-(2-pyridinyl-κ*N*)phenyl-κ*C*][2-[1-(phenylmethyl)-1*H*-1,2,3-triazol-4-yl-κ*N*^3^]pyridine-κ*N*]iridium(1+)hexafluorophosphate(1−) ([Ir(df-ppy)_2_(ptb)](PF_6_)) was synthesised and characterised as previously described.^50^ Solutions were prepared using freshly distilled ACN with 0.1 M TBAPF_6_ unless otherwise stated. DMF was stored on 4 Å molecular sieves (Na_12_[(AlO_2_)_12_(SiO_2_)_12_]·*x*H_2_O; 1.6 mm diameter pellets; Merck, Australia) prior to analysis.^56^ Solutions were degassed with argon prior to analysis, unless otherwise stated.

The ECL cell comprised a cylinder-shaped glass vessel with a custom-built Teflon lid (Fig. S11†), housed in a light-tight Faraday cage. A 3 mm glassy carbon working electrode, a 2 mm platinum disk counter electrode and a leak free Ag/AgCl reference electrode (model KZT-5, 5 mm diameter; Innovative Instruments, USA) were used for all electrochemical and ECL experiments. ECL intensity was measured by an extended range PMT (Electron Tubes model 9828SB) positioned directly under the base of the cell. The PMT was operated at 900 V provided by a power supply (PM20D) and voltage divider (C611, Electron Tubes).

A PGSTAT128N potentiostat (Metrohm Autolab B.V.) was used to apply the working electrode potential and measure the current across the cell, in addition to acquiring the voltage for ECL intensity from the PMT (*via* a transimpedance amplifier) through an auxiliary channel. The potentiostat was fitted with a pX1000 module to measure the potential difference between the counter and reference electrode.

Photographic images were collected by replacing the PMT with a Canon EOS 6D DSLR camera (Canon, Japan) fitted with a Tonika AT-X PRO MACRO 100 mm f/2.8 D lens (Kenko Tonika Co., Japan). ISO values (typically 8000) and aperture (F2.8-F10) were adjusted as required. The brightness of images was further adjusted by software to aid comparison of emission colour. The HSV values for the colour at the working and counter electrodes in the original photographs are tabulated in the ESI (Tables S3–S5†). The camera was activated *via* the Autolab potentiostat configurable DIO port, using a simple transistor switch and relay to control the shutter release (25 s exposure).

## Conclusions

This instrumental approach has provided a new understanding of emerging multiple-luminophore ECL systems, including: the nature of the counter electrode potential under ECL experimental conditions; the emission of ECL at this electrode; a mechanism-based rationalisation of the interactions of different metal-complex luminophores; and a previously unknown ECL pathway for the Ir(ppy)_3_ complex at negative potentials, which occurs even in the absence of TPrA. Although the large negative potentials required to generate this ECL are not normally desired at the working or counter electrodes when using Ir(ppy)_3_ (which has a lower oxidation potential than most commonly used electrochemiluminophores), they may be encountered when this complex is incorporated into multi-colour- or potential-resolved systems, or in less conventional electrochemical cell configurations. Depending on the dimensions and arrangement of the electrodes, the ECL from Ir(ppy)_3_ may occur simultaneously at both electrodes, or in combination with ECL from a second luminophore.

Understanding the electrochemical parameters of both anodic and cathodic electrodes/poles of the ECL instrumental approach in conjunction with the nature of the system from a mechanistic standpoint will spur new advances in multi-colour, potential-resolved and bipolar electrochemistry ECL systems. As the potential at the counter electrode is highly dependent on the reaction conditions and cell configuration, simultaneous spatially resolved quantitative ECL bioassays at the working and counter electrodes would be problematic, and was not the intended rationale for this study. However, we envisage that the selective interaction of different co-reactants with multi-luminophore ECL systems to generate different ECL profiles at the two electrodes at multiple applied potentials could be exploited for rapid qualitative screening (for example of different drug classes).

## Data availability

Supplementary data underpinning this publication are openly available from the University of Strathclyde Knowledge Base at https://doi.org/10.15129/cc9c48e7-b4e2-4366-8236-9cacf018b78a.

## Author contributions

The project was conceptualised by Dr Dennany and Prof. Francis. Exploratory investigations were carried out by Dr Theakstone and Dr Dennany. Subsequent investigations were conducted by Ms Adamson under the supervision of Dr Kerr and Prof. Francis. Throughout the project, additional technical advice and assistance was provided by Dr Soulsby, Dr Doeven and Prof. Hogan. All authors contributed to discussions of experimental design and data interpretation. The manuscript was written by Ms Adamson, Dr Kerr, Dr Dennany and Prof. Francis, and reviewed and edited by all authors. Funding was acquired by Prof. Francis, Dr Dennany and Dr Kerr.

## Conflicts of interest

There are no conflicts to declare.

## Supplementary Material

SC-012-D1SC01236C-s001

## References

[cit1] Chemiluminescence and Bioluminescence: Past, Present and Future, ed. A. Roda, RSC, Cambridge, 2011

[cit2] Yang M., Huang J., Fan J., Du J., Pu K., Peng X. (2020). Chem. Soc. Rev..

[cit3] Analytical Electrogenerated Chemiluminescence: From Fundamentals to Bioassays, ed. N. Sojic, Royal Society of Chemistry, Cambridge, 2020

[cit4] Qi H., Zhang C. (2020). Anal. Chem..

[cit5] Electrogenerated Chemiluminescence, ed. A. J. Bard, Marcel Dekker, New York, 2004

[cit6] Doeven E. H., Barbante G. J., Hogan C. F., Francis P. S. (2015). ChemPlusChem.

[cit7] Shu J., Han Z., Zheng T., Du D., Zou G., Cui H. (2017). Anal. Chem..

[cit8] Wang Y.-Z., Ji S.-Y., Xu H.-Y., Zhao W., Xu J.-J., Chen H.-Y. (2018). Anal. Chem..

[cit9] Soulsby L. C., Doeven E. H., Pham T. T., Eyckens D. J., Henderson L. C., Long B. M., Guijt R. M., Francis P. S. (2019). Chem. Commun..

[cit10] Voci S., Duwald R., Grass S., Hayne D. J., Bouffier L., Francis P. S., Lacour J., Sojic N. (2020). Chem. Sci..

[cit11] Han F., Jiang H., Fang D., Jiang D. (2014). Anal. Chem..

[cit12] Zhou B., Zhu M., Hao Y., Yang P. (2017). ACS Appl. Mater. Interfaces.

[cit13] Kerr E., Doeven E. H., Barbante G. J., Hogan C. F., Hayne D. J., Donnelly P. S., Francis P. S. (2016). Chem. Sci..

[cit14] Miao W. (2008). Chem. Rev..

[cit15] Choi J.-P., Bard A. J. (2005). Anal. Chim. Acta.

[cit16] Zhang J., Kerr E., Usman K. A. S., Doeven E. H., Francis P. S., Henderson L. C., Razal J. M. (2020). Chem. Commun..

[cit17] Soulsby L. C., Hayne D. J., Doeven E. H., Chen L., Hogan C. F., Kerr E., Adcock J. L., Francis P. S. (2018). ChemElectroChem.

[cit18] Theakstone A. G., Doeven E. H., Conlan X. A., Dennany L., Francis P. S. (2019). Chem. Commun..

[cit19] Bruce D., Richter M. M. (2002). Anal. Chem..

[cit20] Doeven E. H., Zammit E. M., Barbante G. J., Hogan C. F., Barnett N. W., Francis P. S. (2012). Angew. Chem., Int. Ed..

[cit21] Barbante G. J., Kebede N., Hindson C. M., Doeven E. H., Zammit E. M., Hanson G. R., Hogan C. F., Francis P. S. (2014). Chem.–Eur. J..

[cit22] Li H., Bouffier L., Arbault S., Kuhn A., Hogan C. F., Sojic N. (2017). Electrochem. Commun..

[cit23] Wang Y.-Z., Xu C.-H., Zhao W., Guan Q.-Y., Chen H.-Y., Xu J.-J. (2017). Anal. Chem..

[cit24] Guo W., Ding H., Gu C., Liu Y., Jiang X., Su B., Shao Y. (2018). J. Am. Chem. Soc..

[cit25] Moghaddam M. R., Carrara S., Hogan C. F. (2019). Chem. Commun..

[cit26] Chen L., Doeven E. H., Wilson D. J. D., Kerr E., Hayne D. J., Hogan C. F., Yang W., Pham T. T., Francis P. S. (2017). ChemElectroChem.

[cit27] Suzuki K., Kobayashi A., Kaneko S., Takehira K., Yoshihara T., Ishida H., Shiina Y., Oishi S., Tobita S. (2009). Phys. Chem. Chem. Phys..

[cit28] Tsuboyama A., Iwawaki H., Furugori M., Mukaide T., Kamatani J., Igawa S., Moriyama T., Miura S., Takiguchi T., Okada S., Hoshino M., Ueno K. (2003). J. Am. Chem. Soc..

[cit29] Juris A., Balzani V., Barigelletti F., Campagna S., Belser P., Von Zelewsky A. (1988). Coord. Chem. Rev..

[cit30] Flamigni L., Barbieri A., Sabatini C., Ventura B., Barigelletti F. (2007). Top. Curr. Chem..

[cit31] Laser D., Bard A. J. (1975). J. Electrochem. Soc..

[cit32] Bard A. J. and Faulkner L. R., Electrochemical Methods: Fundamentals and Applications, John Wiley & Sons, New York, 2nd edn, 2001

[cit33] Chen L., Hayne D. J., Doeven E. H., Agugiaro J., Wilson D. J. D., Henderson L. C., Connell T. U., Nai Y. H., Alexander R., Carrara S., Hogan C. F., Donnelly P. S., Francis P. S. (2019). Chem. Sci..

[cit34] Juris A., Balzani V., Belser P., von Zelewsky A. (1981). Helv. Chim. Acta.

[cit35] Kapturkiewicz A., Angulo G. (2003). Dalton Trans..

[cit36] Lai R. Y., Bard A. J. (2003). J. Phys. Chem. A.

[cit37] Smith P. J., Mann C. K. (1969). J. Org. Chem..

[cit38] Noffsinger J. B., Danielson N. D. (1987). Anal. Chem..

[cit39] Miao W., Choi J.-P., Bard A. J. (2002). J. Am. Chem. Soc..

[cit40] Laboratory Techniques in Electroanalytical Chemistry, ed. P. Kissinger and W. R. Heineman, CRC Press, New York, 2nd edn, 1996

[cit41] Kerr E., Doeven E. H., Wilson D. J. D., Hogan C. F., Francis P. S. (2016). Analyst.

[cit42] Doeven E. H., Zammit E. M., Barbante G. J., Francis P. S., Barnett N. W., Hogan C. F. (2013). Chem. Sci..

[cit43] Pons S., Khoo S. B. (1982). Electrochim. Acta.

[cit44] Folley J. K., Korzeniewski C., Pons S. (1988). Can. J. Chem..

[cit45] Zieja J., Gadomska-Trzos J., Stojek Z. (2001). Electroanalysis.

[cit46] Doeven E. H., Barbante G. J., Kerr E., Hogan C. F., Endler J. A., Francis P. S. (2014). Anal. Chem..

[cit47] Barbante G. J., Hogan C. F., Wilson D. J. D., Lewcenko N. A., Pfeffer F. M., Barnett N. W., Francis P. S. (2011). Analyst.

[cit48] Kerr E., Doeven E. H., Barbante G. J., Hogan C. F., Bower D., Donnelly P. S., Connell T. U., Francis P. S. (2015). Chem. Sci..

[cit49] Barbante G. J., Doeven E. H., Kerr E., Connell T. U., Donnelly P. S., White J. M., Lópes T., Laird S., Hogan C. F., Wilson D. J. D., Barnard P. J., Francis P. S. (2014). Chem.–Eur. J..

[cit50] Soulsby L. C., Hayne D. J., Doeven E. H., Wilson D. J. D., Agugiaro J., Connell T. U., Chen L., Hogan C. F., Kerr E., Adcock J. L., Donnelly P. S., White J. M., Francis P. S. (2018). Phys. Chem. Chem. Phys..

[cit51] Soulsby L. C., Agugiaro J., Wilson D. J. D., Hayne D. J., Doeven E. H., Chen L., Pham T. T., Connell T. U., Driscoll A. J., Henderson L. C., Francis P. S. (2020). ChemElectroChem.

[cit52] Muegge B. D., Richter M. M. (2004). Anal. Chem..

[cit53] Zu Y., Bard A. J. (2000). Anal. Chem..

[cit54] Valenti G., Fiorani A., Li H., Sojic N., Paolucci F. (2016). ChemElectroChem.

[cit55] Kerr E., Alexander R., Francis P. S., Guijt R. M., Barbante G. J., Doeven E. H. (2021). Front. Chem..

[cit56] Armarego W. L. F. and Chai C. L. L., Purification of Laboratory Chemicals, Butterworth-Heinemann, Oxford, 6th edn, 2009

