# Supplementary material for: Emission from the working and counter electrodes under co-reactant electrochemiluminescence conditions
Source: Chem Sci. 2021 Jun 25;12(28):9770–7. doi: 10.1039/d1sc01236c (PMC8293983; doi:10.1039/d1sc01236c)
Supplement: SC-012-D1SC01236C-s001 [file SC-012-D1SC01236C-s001.pdf]

Electronic Supplementary Material (ESI) for Chemical Science.
This journal is © The Royal Society of Chemistry 2021

Electronic Supplementary Information

Emission from the working and counter electrodes under co-reactant electrochemiluminescence conditions

Natasha S. Adamson, Ashton G. Theakstone, Lachlan C. Soulsby, Egan H. Doeven, Emily Kerr, Conor F. Hogan, Paul S. Francis, and Lynn Dennany

Table S1. Selected properties of $[Ru(bpy)_2(L)]^{2+}$ complexes in acetonitrile.[1]

	Abs.[a]	PL (*r.t.*)[b]	Electrochemical potentials			ECL[c]
L[d]	λ_{max} / nm	λ_{max} / nm	$E^{0\prime}$ (ox) / V *vs* $Fc^{+/0}$	$E^{0\prime}$ (red) / V *vs* $Fc^{+/0}$	ΔE / eV	ϕ_{ECL} / %
bpy	451	618	0.892	–1.754	2.646	5.0
Me-Ala-bpy-dc	469	668	0.976	–1.467	2.443	6.5
dm-bpy-dc	478	686	0.983	–1.396	2.379	4.9

[a]Absorbance maxima in visible region. [b]Corrected for the change in instrument sensitively over the examined wavelength range. [c]Annihilation ECL efficiency relative to $[Ru(bpy)_3]^{2+}$ = 5.0%.[2] [d]Ligands: 2,2′-bipyridine (bpy), *N*[4],*N*[4′]-bis((2*S*)-1-methoxy-1-oxopropan-2-yl) 2,2′-bipyridyl-4,4′-dicarboxamide (Me-Ala-bpy-dc), and dimethyl 2,2′-bipyridine-4,4′-dicarboxylate (dm-bpy-dc).

Table S2. Estimates of the excited state (^{3}MLCT) energy (E_{00}) for $[Ru(bpy)_2(L)]^{2+}$, and corresponding free energy (ΔG) for the reaction: $[Ir(ppy)_3]^+ + [Ru(bpy)_2(L)]^+ \rightarrow Ir(ppy)_3 + [Ru(bpy)_2(L)]^{2+*}$.

L[a]	λ_{max} (*r.t.*)[b] / eV	ΔG[c] / eV	λ_{max} (77 K) / eV	ΔG[d] / eV
bpy	2.01	–0.08	2.14[e]	+0.05
Me-Ala-bpy-dc	1.86	+0.06	1.99[f]	+0.19
dm-bpy-dc	1.81	+0.08	1.94[f]	+0.21

[a]Ligands: 2,2′-bipyridine (bpy), *N*[4],*N*[4′]-bis((2*S*)-1-methoxy-1-oxopropan-2-yl) 2,2′-bipyridyl-4,4′-dicarboxamide (Me-Ala-bpy-dc), and dimethyl 2,2′-bipyridine-4,4′-dicarboxylate (dm-bpy-dc). [b]From reference [1]. [c]Estimated using $\Delta G = E^{0\prime}(\text{red}) - E^{0\prime}(\text{ox}) + E_{00}$, where E_{00} is approximated by the λ_{max} of the photoluminescence emission at room temperature. [d]Estimated using $\Delta G = E^{0\prime}(\text{red}) - E^{0\prime}(\text{ox}) + E_{00}$, where E_{00} is approximated by the λ_{max} of the photoluminescence emission at 77 K. [e]From reference [3]. [f]Estimated from the λ_{max} of $[Ru(bpy)_3]^{2+}$ and the difference in the λ_{max} between $[Ru(bpy)_2(L)]^{2+}$ and $[Ru(bpy)_3]^{2+}$ at room temperature.

The λ_{max} at 77 K should provide a more accurate measure of E_{00} than the λ_{max} at room temperature. These first approximations of ΔG from $E^{0\prime}$ (red) – $E^{0\prime}$ (ox) + E_{00}, however, neglect electrostatic interactions and entropic contributions.[4] The observation of ECL from electrogenerated $[Ir(ppy)_3]^+$ and $[Ru(bpy)_3]^+$ species[5] indicates that E_{00} derived from spectra obtained at ambient temperature may incidentally compensate for other factors and give a more accurate approximation of ΔG for the above reaction with this series of closely related complexes.

Table S3. HSV values for the colour at the working and counter electrodes in the original photographs for Figure 2.

	(a) $[Ru(bpy)_3]^{2+}$			(b) $Ir(ppy)_3$			
CE	**19**<br>100<br>82		**H**<br>S<br>B	**126**<br>64<br>65	**129**<br>64<br>70		**H**<br>S<br>V
WE		**24**<br>100<br>98	**H**<br>S<br>B	**120**<br>80<br>14		**126**<br>56<br>67	**H**<br>S<br>V
	-2.7 V	+1.4 V		-2.5 V	-2.2 V	+0.7 V	

Table S4. HSV values for the colour at the working and counter electrodes in the original photographs for Figure 5.

CE	**24**<br>100<br>88	**52**<br>39<br>81	**104**<br>43<br>80	**131**<br>75<br>8		**H**<br>S<br>V
WE	**127**<br>79<br>10	**119**<br>61<br>7	**97**<br>68<br>3	**115**<br>56<br>47	**16**<br>100<br>64	**H**<br>S<br>V
	-2.5 V	-2.4 V	-2.3 V	+0.7 V	+1.4 V	

Table S5. HSV values for the colour at the working and counter electrodes in the original photographs for Figure 7.

CE	**204**<br>65<br>28	**126**<br>52<br>80	**129**<br>59<br>69			**H**<br>S<br>V
WE	**116**<br>50<br>74	**107**<br>49<br>38		**127**<br>59<br>70	**199**<br>67<br>96	**H**<br>S<br>V
	-2.6 V	-2.5 V	-1.8 V	+0.7 V	+1.6 V	

Figure S1. (a,b) The plots of Fig. 1b and 1c extended over three cyclic voltametric scans.

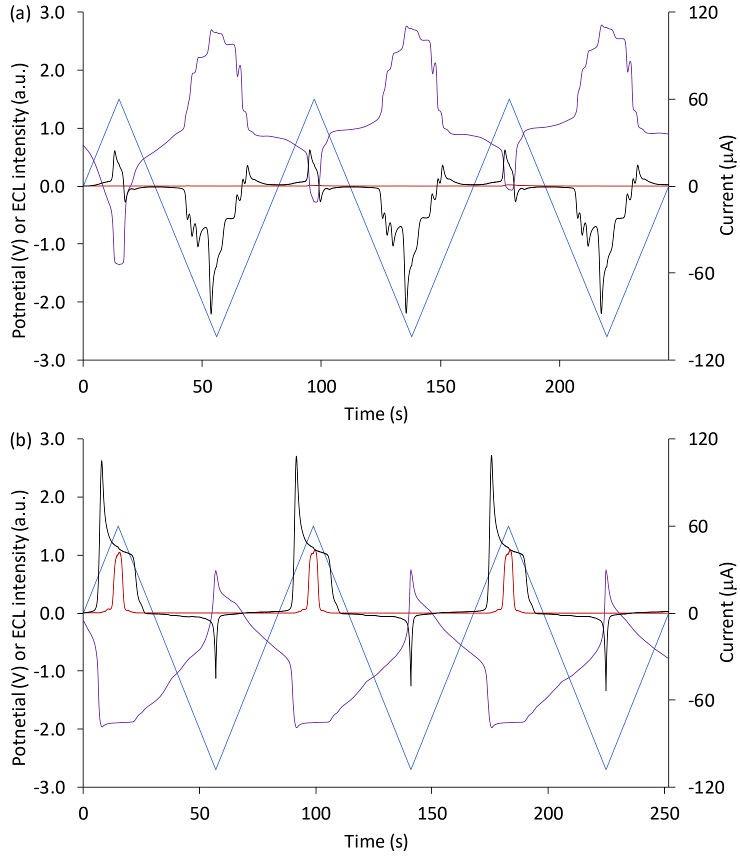

Figure S2. (a) Cyclic voltammograms (black plots) and corresponding ECL intensities (red plots) for 5 μM $[Ru(bpy)_3]^{2+}$ with 10 mM TPrA (solid plots) and 1 mM $[Ru(bpy)_3]^{2+}$ (dashed plots). (b) Counter electrode potentials (black plots) and corresponding ECL intensities (red plots) for 5 μM $[Ru(bpy)_3]^{2+}$ with 10 mM TPrA (solid plots) and 1 mM $[Ru(bpy)_3]^{2+}$ (dashed plots). All solutions contain 0.1 M $TBAPF_6$ electrolyte in ACN and were degassed for 10 min prior to analysis (scan rate 0.1 V/s).

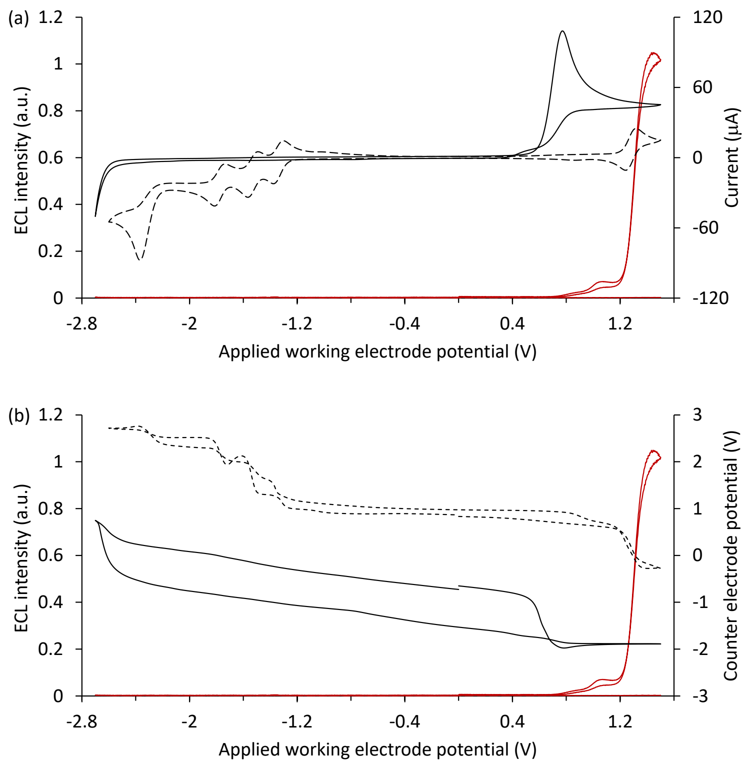

Figure S3. (a) Voltage at the counter electrode and (b) current between the working and counter electrode upon application of a chronoamperometric pulse at –2.5 V (blue plot), –2.6 V (orange plot), –2.7 V (green plot) and –2.8 V (yellow plot), *vs* Ag/AgCl, for a solution containing 5 μM $[Ru(bpy)_3]^{2+}$ and 10 mM TPrA co-reactant, in acetonitrile with 0.1 M $TBAPF_6$ electrolyte.

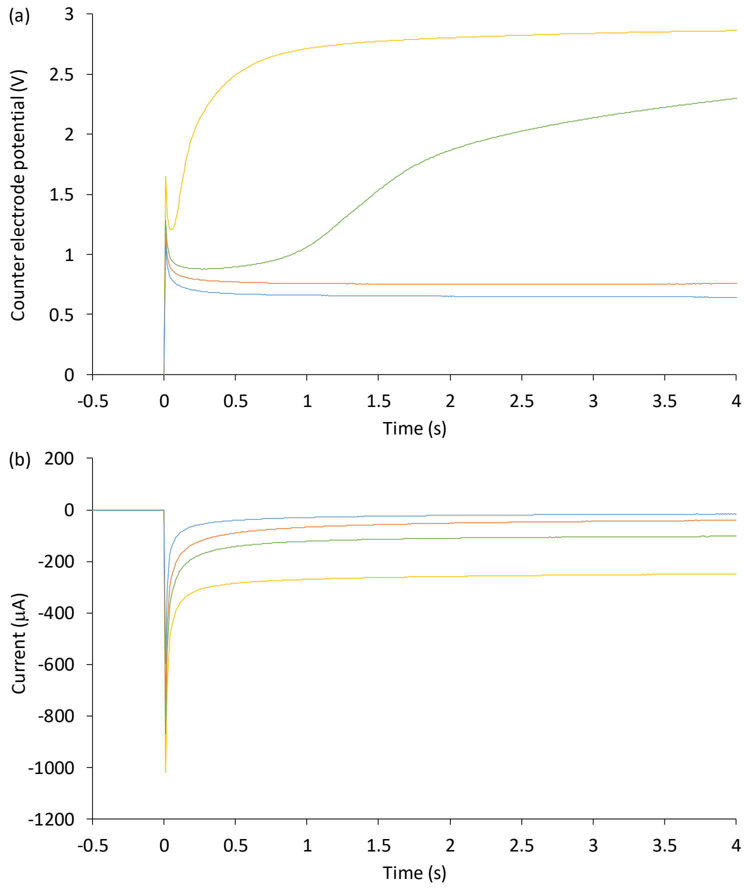

Figure S4. Current (black plots), potential measured at the counter electrode (purple plots), and ECL intensity (green plots), over time, when scanning (a) positive or (b) negative potentials (blue plots) at 0.1 V/s, for 0.2 mM $Ir(ppy)_3$ with (solid plots) and without (dashed plots) 10 mM TPrA, with 0.1 M $TBAPF_6$ electrolyte in ACN. To show the plots on the same scales, the current for 0.2 mM $Ir(ppy)_3$ with 10 mM TPrA at positive potentials (*) was divided by five. ECL intensities were multiplied by five (†) or fifty (‡).

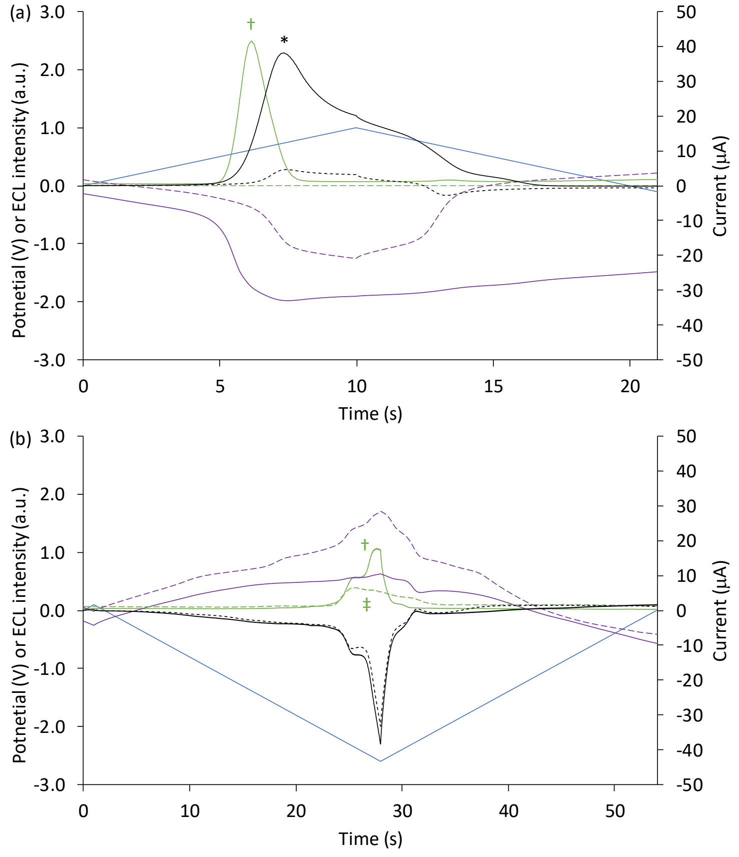

Figure S5. (a,b) The plots of Fig. S3a and S3b over three cyclic voltametric scans.

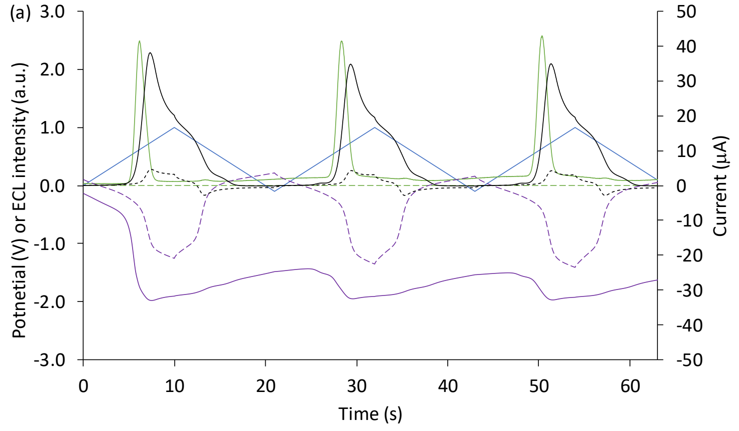

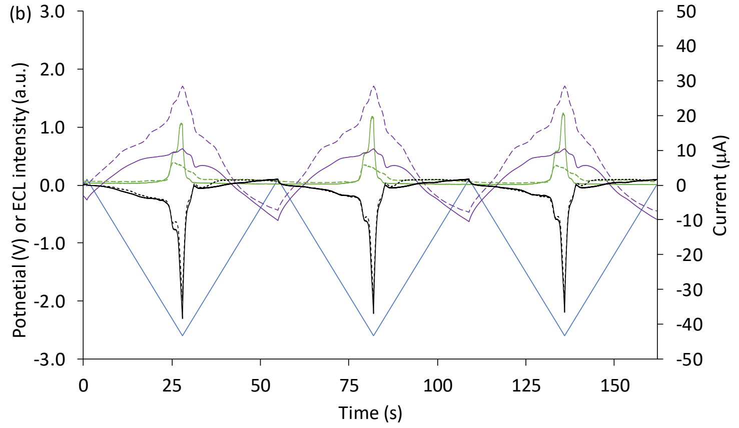

Figure S6. (a,b) Counter electrode potential (black plots) and ECL intensities (green plots) for 0.2 mM $Ir(ppy)_3$ with (solid plots) and without (dashed plots) 10 mM TPrA, in ACN with 0.1 M $TBAPF_6$, when applying (a) positive or (b) negative potentials at the working electrode. For clarity, the ECL intensity in the absence of TPrA at negative potentials (†) was multiplied by 10.

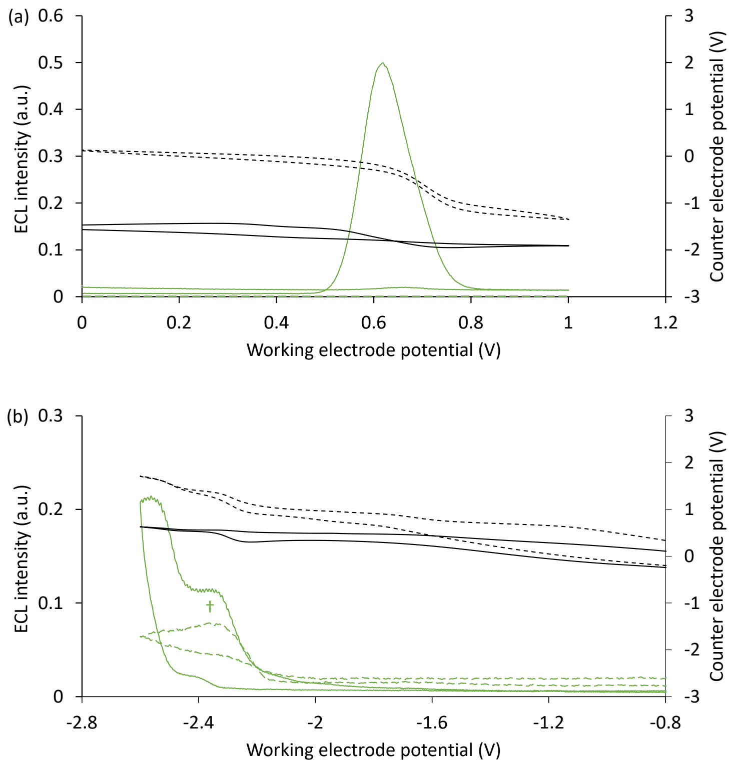

Figure S7. Counter electrode potential (black plot) and ECL intensity (orange plot) for 0.1 mM $Ir(ppy)_3$ and 5 μM $[Ru(bpy)_3]^{2+}$ with 10 mM TPrA, in ACN with 0.1 M $TBAPF_6$.

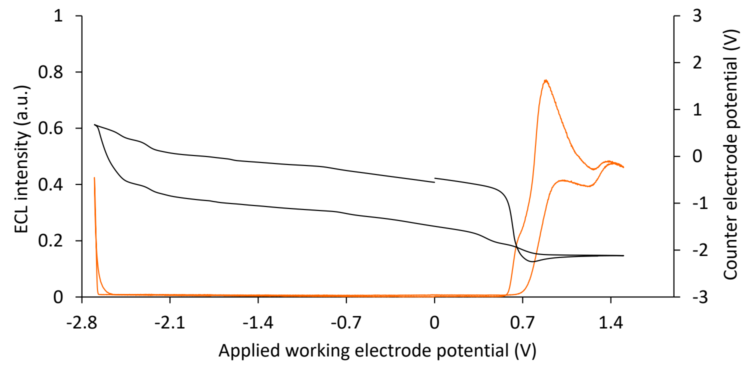

Figure S8. Photograph of the ECL of 5 μM $[Ru(bpy)_3]^{2+}$, 0.1 mM $Ir(ppy)_3$ and 10 mM TPrA, with 0.1 M $TBAPF_6$ in acetonitrile at the working electrode upon application of 0.9 V *vs* Ag/AgCl.

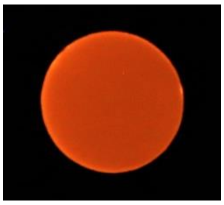

Figure S9. (a) Counter electrode potential (black plot) and ECL intensity (green plot) for 0.1 mM $Ir(ppy)_3$ and 40 μM $[Ir(df\text{-}ppy)_2(ptb)]^+$ with 10 mM TPrA, in ACN with 0.1 M $TBAPF_6$.

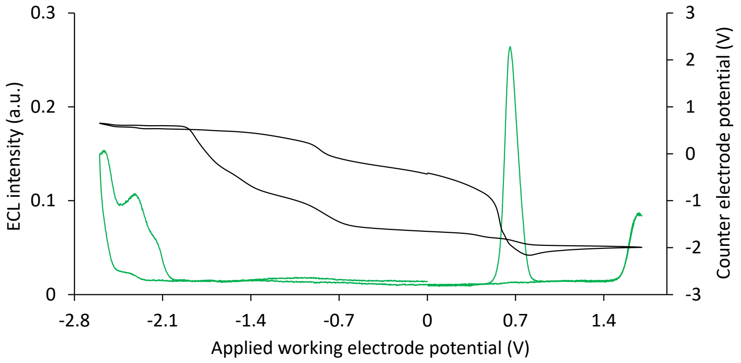

Figure S10. Applied potential (blue plots), current (black plots), potential measured at the counter electrode (purple plots), and ECL intensity (orange plots), over time, when scanning to -2.7 V (solid plots) or -2.9 V (dashed plots) at 0.1 V/s, for 0.1 mM $Ir(ppy)_3$, 5 μM $[Ru(bpy)_3]^{2+}$ and 10 mM TPrA, with 0.1 M $TBAPF_6$ electrolyte in ACN.

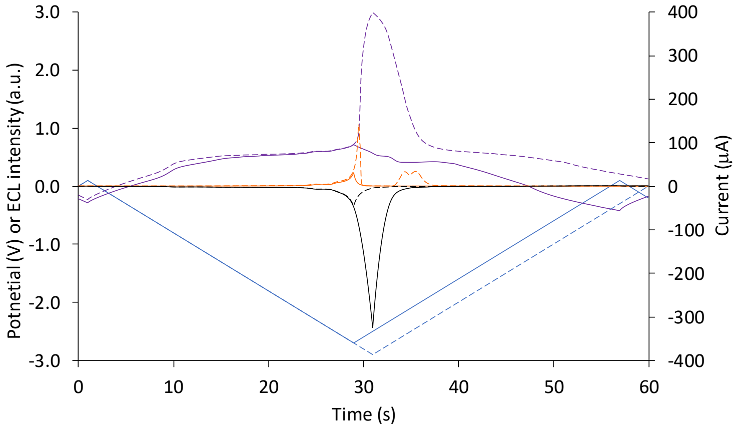

Figure S11. Sectional view of the ECL cell configuration.

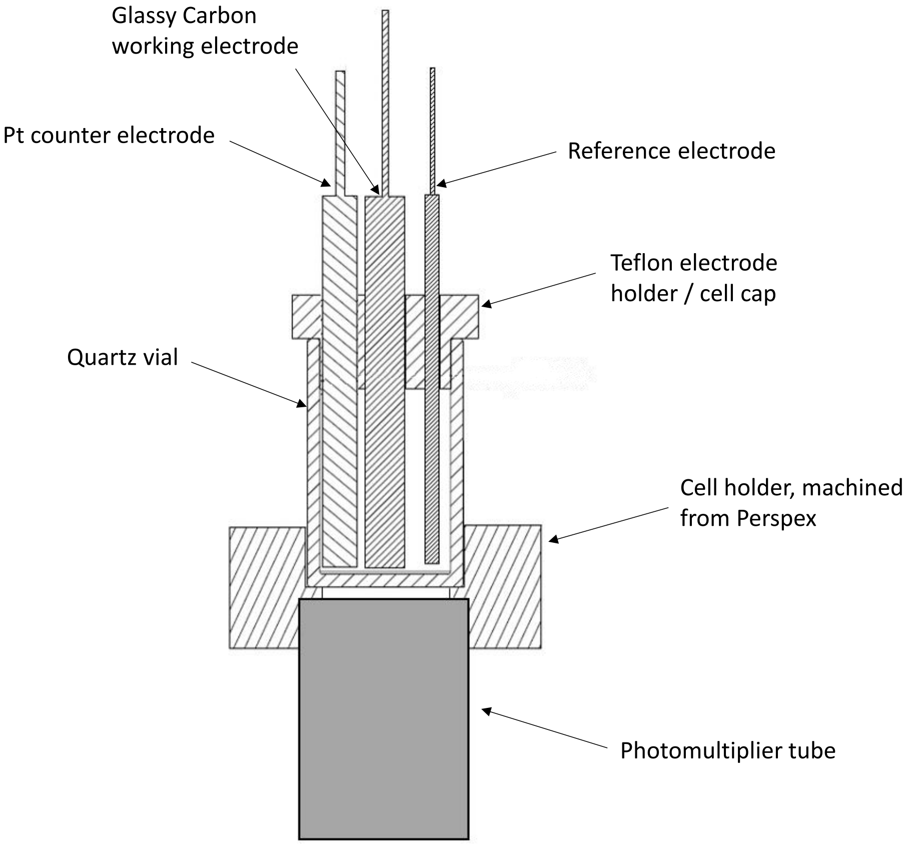

Figure S12. (a,b) Cyclic voltammograms (black plots) and corresponding ECL intensities (green plots) in DMF for 0.2 mM Ir(ppy)$_3$ with (solid plots) and without (dashed plots) 10 mM TPrA, when applying (a) positive or (b) negative potentials at the working electrode. All solutions contained 0.1 M $TBAPF_6$ electrolyte and were degassed for 10 min prior to analysis (scan rate 0.1 V/s). For clarity, the current for 0.2 mM Ir(ppy)$_3$ with 10 mM TPrA at positive potentials (*) was divided by eight, and the ECL intensity in the absence of TPrA at negative potentials (†) was multiplied by 5. The inset photograph shows the ECL at the working electrode from Ir(ppy)$_3$ in the absence of TPrA, at an applied potential of -2.5 V.

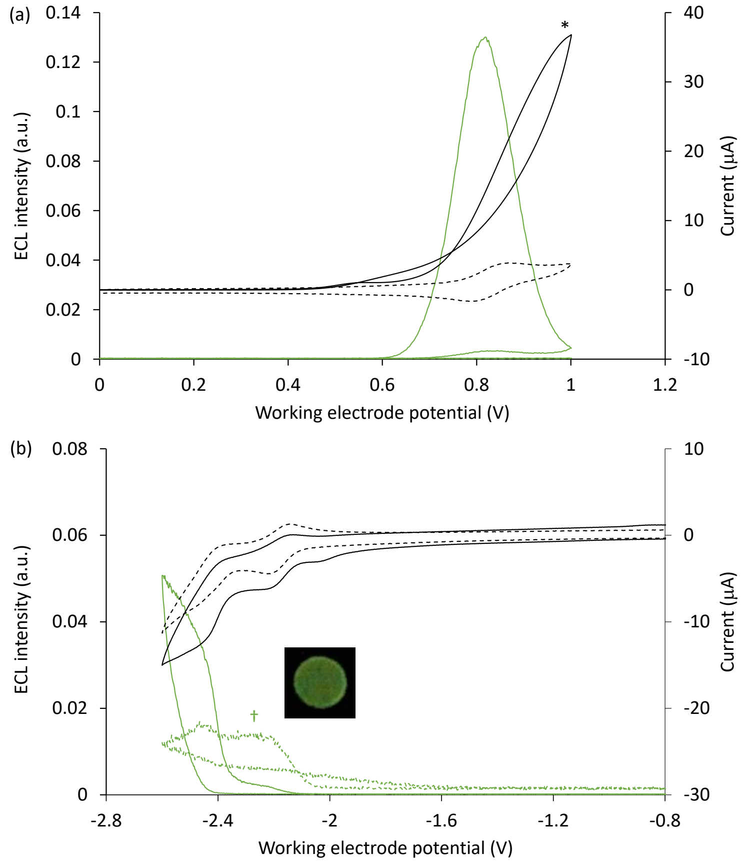

References

1. G. J. Barbante, C. F. Hogan, D. J. D. Wilson, N. A. Lewcenko, F. M. Pfeffer, N. W. Barnett and P. S. Francis, *Analyst*, 2011, **136**, 1329-1338.
2. W. L. Wallace and A. J. Bard, *J. Phys. Chem.*, 1979, **83**, 1350-1357.
3. L. Chen, E. H. Doeven, D. J. D. Wilson, E. Kerr, D. J. Hayne, C. F. Hogan, W. Yang, T. T. Pham and P. S. Francis, *ChemElectroChem*, 2017, **4**, 1797-1808.
4. A. Kapturkiewicz, *Adv. Electrochem. Sci. Eng.*, 1997, **5**, 1-60.
5. E. Kerr, E. H. Doeven, G. J. Barbante, C. F. Hogan, D. Bower, P. S. Donnelly, T. U. Connell and P. S. Francis, *Chem. Sci.*, 2015, **6**, 472-479.
